# Mitochondrial Genetic Diversity, Population Structure and Detection of Antillean and Amazonian Manatees in Colombia: New Areas and New Techniques

**DOI:** 10.3389/fgene.2021.726916

**Published:** 2021-11-26

**Authors:** Susana Caballero, Maria Camila Ortiz-Giral, Laura Bohorquez, Juan Diego Lozano Mojica, Dalila Caicedo-Herrera, Katherine Arévalo-González, Antonio A. Mignucci-Giannoni

**Affiliations:** ^1^ Laboratorio de Ecología Molecular de Vertebrados Acuáticos (LEMVA), Departamento de Ciencias Biológicas, Universidad de Los Andes, Bogotá, Colombia; ^2^ Fundación Omacha, Bogotá, Colombia; ^3^ Cabildo Verde, Sabana de Torres, Colombia; ^4^ Fundación Internacional para La Defensa de La Naturaleza y La Sustentabilidad-FINS, Chetumal, Mexico; ^5^ Centro de Conservación de Manatíes del Caribe, Universidad Interamericana de Puerto Rico, Bayamón, Puerto Rico; ^6^ Center for Conservation Medicine and Ecosystem Health, Ross University School of Veterinary Medicine, Basseterre, St. Kitts

**Keywords:** Colombia, *Trichechus manatus*, *Trichechus inunguis*, mtDNA, environmental DNA, population structure

## Abstract

The Antillean manatee (*Trichechus manatus*) and the Amazonian manatee (*Trichechus inunguis*) are distributed in rivers in the Caribbean and Amazonian region of Colombia respectively. For 30 years, genetic information has been obtained from these populations in order to inform conservation programs for these endangered species and decide on the location to release them back to the wild. However, in previous studies, samples from rivers in some areas of the country were not included, given the difficulties to access these regions due to either logistic or safety issues. In this study, we analyzed mitochondrial DNA (mtDNA) control region (CR) sequences of from samples of *T. manatus* (*n* = 37) and *T. inunguis* (*n* = 4) (410 and 361 bp, respectively), obtained in new and previously unexplored rivers and bays in the country, including Santa Marta, Urabá Gulf, Ayapel Marsh (San Jorge River Basin), Meta River and Magdalena Medio and the low Magdalena River (Cesar Province and Canal del Dique) as well as additional samples from Puerto Nariño in the Colombian Amazon. Our results included the discovery of two newly described mtDNA CR haplotypes for *T. manatus*. In addition, we confirmed significant population differentiation at the mitochondrial level between the Magdalena and Sinú rivers and differentiation among areas of the same river, including the middle and low Magdalena River. This differentiation may be related to anthropic changes in the river since construction of the Canal del Dique in the XVI century. We also tested environmental DNA sampling and analyses techniques to evaluate its potential use for manatee detection and monitoring in bodies of water in Colombia, in order to evaluate new areas for future manatee conservation initiatives. We emphasize the need to continue using genetic information to provide evidence on the potential best locations to undertake animal release to prevent outbreeding depression.

## Introduction

Manatees belong to the order Sirenia, and the family Trichechidae, with three species found in tropical areas of the world; two in the American continent, the West Indian manatee (*Trichechus manatus*) and the Amazonian manatee (*Trichechus inunguis*), and one in Africa (*Trichechus senegalensis*). The West Indian manatee is distributed from Florida to Northeastern Brazil, and subdivided into the Florida subspecies (*T. manatus latirostris*) and the Antillean subspecies (*T. manatus manatus*) (Domning and Hayek 1986). The Antillean subspecies inhabits the Greater Antilles, the Caribbean coast of Central and South America, the Guyanas, and Brazil (Self-Sullivan and Mignucci-Giannoni 2008). The Amazonian manatee lives in the rivers and tributaries of the Amazon Basin (Marmontel et al., 2016).

The IUCN categorized the Antillean manatee as endangered (Self-Sullivan and Mignucci-Giannoni 2008) and the Amazonian manatee as vulnerable (Marmontel et al., 2016), with populations of both species in a decreasing trend, being affected by habitat destruction, direct takes, and negative fisheries interactions. In Colombia, both species are considered endangered (Trujillo et al., 2006a; Trujillo et al., 2006b) and are protected by law and included in national management plans for more than 15 years (Trujillo et al., 2013). In addition, conservation initiatives have successfully included local communities in monitoring programs for manatees and in rehabilitation programs for stranded or orphaned manatees with high success rates in releasing these animals back to their natural habitat (Trujillo et al., 2013). Interestingly, Antillean manatees in Colombia are mainly found in rivers of the Magdalena, Sinú, San Jorge, Atrato, and Orinoco Basins, with some individuals from particular populations close to the coast moving to marine habitats for reduced amounts of time and going back to the freshwater habitats (Caicedo-Herrera et al., 2013).

These conservation initiatives have benefited from population genetic information to make informed decisions regarding release areas for rehabilitated animals. The population genetic makeup of the Colombian Antillean manatees has been studied for 30 years, revealing high genetic diversity in the mitochondrial DNA (García-Rodríguez et al., 1998; Vianna et al., 2006; Satizábal et al., 2012), the highest when compared to populations from North America, Caribbean, Central America, Guyanas and Brazil, and slightly lower genetic diversities when analyzing nuclear microsatellite loci (Satizábal et al., 2012). Also, populations appear to be structured depending on their rivers of origin, suggesting female philopatry and male-mediated gene flow (Satizábal et al., 2012). The information for the Amazonian manatees is scarce since access to samples has been limited. However, initial results suggest relatively high mitochondrial genetic diversity but reduced nuclear genetic diversity (Satizábal et al., 2012).

Although advances have been made regarding understanding the genetic component of the two manatee species in Colombia, many questions remain regarding their genetic diversity and phylogeographic patterns in river basins that have not been previously sampled due to logistics or safety difficulties (i.e., Atrato River Basin). Also, discovery and monitoring previously undetected manatee groups is a priority for establishing new local conservation initiatives in the country. Analysis of environmental DNA (eDNA) appears as a fundamental technique (Ruppert et al., 2019) that allows rapid biodiversity monitoring (Deiner et al., 2017). DNA from every organism is released continuously into its environment (soil, water, or air) through the skin, saliva, scales, feces, urine, mucus, or blood (Li et al., 2020). However, in aquatic environments, DNA remains in the water for a limited time**.** For that reason, eDNA analysis provides us with a portrait of the biological community found at a specific site at a specific time (Bakker et al., 2017). Environmental DNA has been previously used successfully for manatee detection in locations from Florida (*Trichechus manatus latirostris*), Cuba (*Trichechus manatus*) and Cameroon (*Trichechus senegalensis*) (Hunter et al., 2018).

This work aims to develop genetic analyses that include previously unsampled populations in Colombia and evaluate the potential use of eDNA technology to detect and monitor manatee populations around the country, as a tool to identify particular locations where to start new manatee conservation initiatives around the country.

## Methodology

### Sampling Locations

**FIGURE 1:**
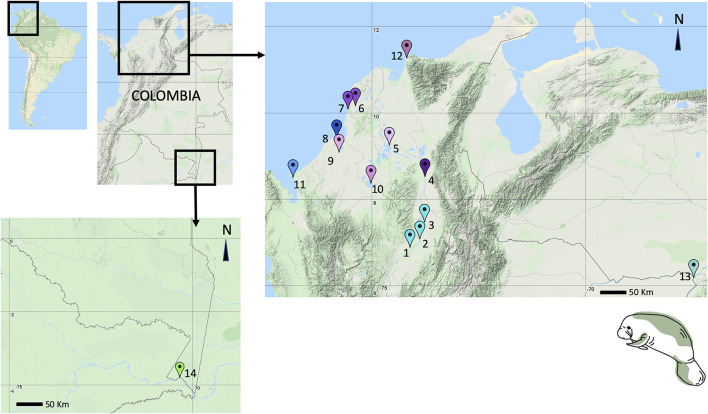
Antillean and Amazonian manatee tissue sampling sites (new to this study): 1. Totumo Marsh, Yondo, Antioquia, 2. San Silvestre Marsh, 3. Paredes Marsh, 4. Aguachica, Cesar, 5. Magangue, 6. Canal del Dique, 7. EL Corchal, Bolivar, 8. Cispatá Bay, 9. Lorica Marsh, 10. Ayapel Marsh, 11. Necocli, Urabá Gulf, Antioquia, 12. Santa Marta, Magdalena, 13. Meta River, 14. Puerto Nariño, Amazonas. Colors in the map refer to colors in the haplotype networks.

For both manatee species, skin samples (*T. manatus* = 35 and *T. inunguis* = 4) were obtained from free-range, live or dead stranded, or captive animals in rehabilitation programs in 14 locations around Colombia (Figure 1, Supplementary Table S1) between 2014 and 2020. Most of these sampling locations included sites where no previous samples had been obtained or no previous sampling efforts had been conducted, including Santa Marta, Morrosquillo Gulf, Urabá Gulf, Canal del Dique, Ayapel Marsh, and Cesar Province. Also, new samples were obtained from locations with previously studied populations (Vianna et al., 2006; Satizábal et al., 2012), such as the Medio and Bajo Magdalena River Basin (Magdalena River and Paredes Marsh), Sinú River Basin, San Jorge River Basin, Orinoco River Basin (Orinoco and Meta rivers), and Puerto Nariño (Amazon River Basin).

### DNA Extraction, Amplification, and Sequencing

DNA was extracted using the Quick-DNA Miniprep kit from Zymo Research (see manufacturer instructions). DNA concentrations were checked on a nanodrop and ran on a 0.8% agarose gel to evaluate DNA quality. Approximately 410 bp of the mitochondrial control region (mtDNA CR) were amplified using the primers CR-4 and CR-5 and following the protocols from Satizábal et al. (2012) and Vianna et al. (2006). Successfully amplified PCR products were cleaned using magnetic beads and sequenced on an ABI 3500 at Universidad de los Andes.

### mtDNA Data Analyses

Sequences were manually edited and aligned using Geneious Prime (www.geneious.com). In order to detect potential new haplotypes among the new samples included in this study, comparisons with haplotypes defined by García-Rodríguez et al. (1998), Cantanhede et al. (2005), Vianna et al. (2006), Satizábal et al. (2012),Lima et al. (2019), Vilaça et al. (2019), and Luna et al. (2021). For *T. manatus*, these included sequences obtained from Florida, Cuba, Dominican Republic, Puerto Rico, Mexico, Belize, Panama, Colombia, Venezuela, French Guiana, and Brazil. For *T. inunguis*, these included samples from the Peruvian Amazon and the Brazilian Amazon. For *T. manatus*, the whole 410 bp sequence length was used for comparisons, and sequences from samples in this study were combined with sequences previously obtained by Satizábal et al. (2012). For *T. inunguis*, sequences were trimmed to 361 bp in order to be able to achieve comparisons with sequences from previous studies, sequences from samples in this study were combined with sequences previously obtained by Satizábal et al. (2012). Haplotypes were defined using MacClade (Maddison and Maddison, 2000). Haplotype networks were constructed using the statistical parsimony methodology from the software TCS Vs 1.21 (Clement et al., 2000) as implemented in the software PopArt (Leigh and Bryant, 2015). For *T. manatus*, two haplotype networks were built, one that included only haplotypes found in Colombian sampling sites (this study and Satizábal et al., 2012) and a second one including haplotypes from Colombian sampling sites plus haplotypes found in Florida, the Caribbean, Central, and South America. For *T. inunguis*, one haplotype network was built, including all haplotypes from the Colombian Amazon (this study plus Satizábal et al., 2012) plus haplotypes from the Peruvian and Brazilian Amazon samples. For *T. manatus*, population structure analyses were carried out following a two-step approach. In the first approach, only Colombian sampling locations were used to determine genetic differentiation among them. The second approach compared among Colombian sampling locations with all other locations from Florida, the Caribbean, Central and South America included in previous genetic analyses. For *T. inunguis*, comparisons were carried out among Colombian Amazon locations and Peruvian and Brazilian Amazon locations. All these genetic differentiation analyses were done using an analysis of molecular variance (AMOVA) as implemented in Arlequin (Excoffier and Lisher, 2010) based on conventional *FST* and *Φ*_ST_ statistics, using 10,000 random permutations. Haplotype (*h*) and nucleotide (*π*) diversity calculations were performed in the program Arlequin Vs 3.5 (Excoffier and Lisher, 2010).

### Environmental DNA Sampling, Laboratory Procedures, and Bioinformatic Analyses

**FIGURE 2:**
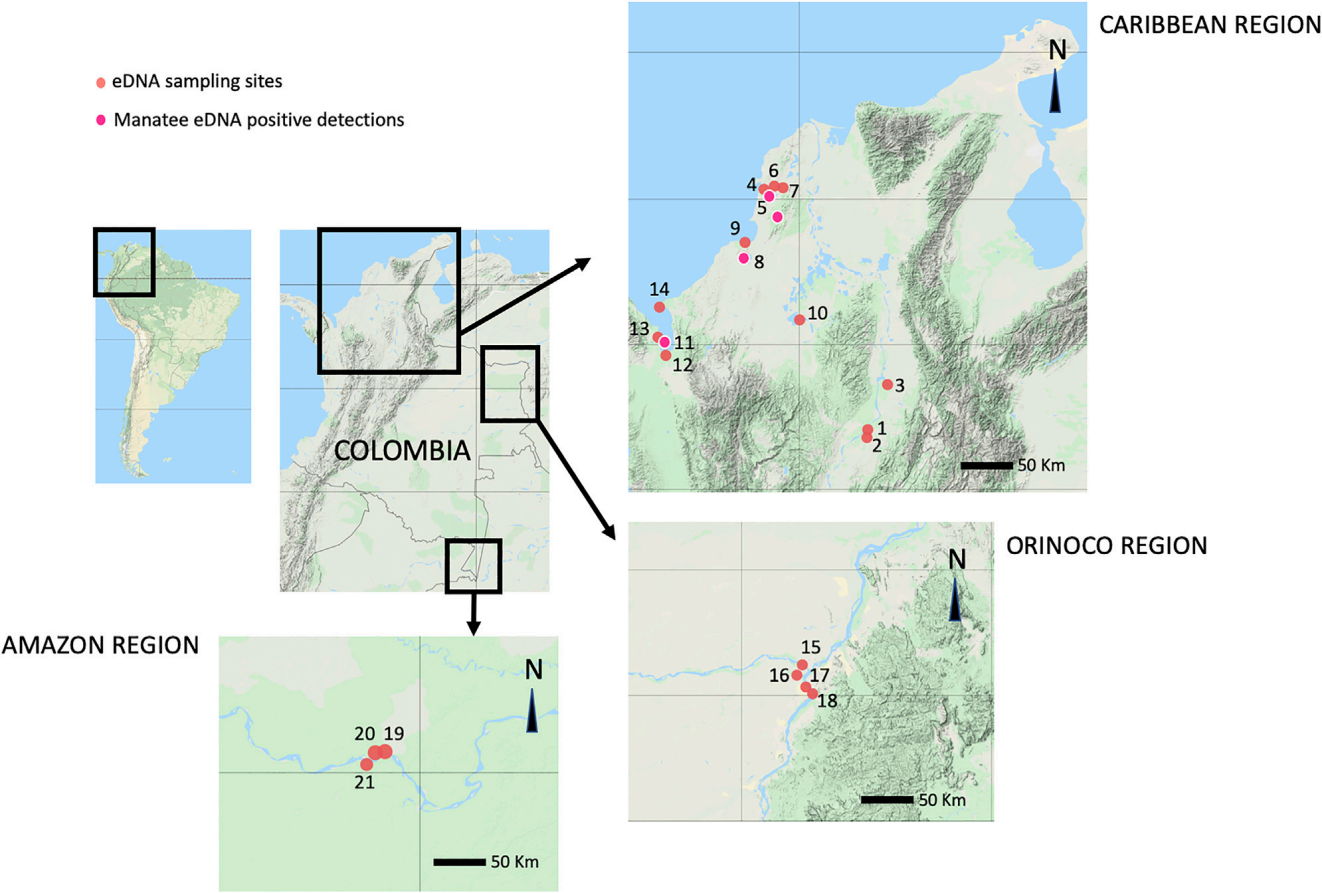
Environmental DNA sampling sites: 1. Chucuri Marsh, 2. San Juan River, 3. Paredes Marsh, 4. De las Flores Marsh, 5. Canal del Dique, 6. EL Floral Marsh, 7. Batallion Lake, 8. Lorica Marsh, 9. Cispata Bay, 10. Ayapel Marsh, 11. Atrato River mouth, 12. Suriqui River mouth, 13. Marriaga Marsh, 14. Rio Negro Cove, 15. Meta River, 16. Vita River, 17. Orinoco River, 18. Bojonawi Reserve, 19. Puerto Nariño, 20. Tarapoto Lake, 21. Caballo Cocha Lake. In bright pink, manatee eDNA positive detection sites: 5. Canal del Dique, 7. Batallion Lake, 8. Lorica Marsh, 11. Atrato River mouth.

Between July 2019 and February 2020, fieldwork was conducted in the Magdalena, San Jorge, Sinú, Atrato, Orinoco, and Amazon river basins, and the Morrosquillo and Urabá gulfs, in order to collect water samples to test the feasibility of eDNA analyses to detect the presence of both manatee species in 21 sampling locations (Figure 2, Supplementary Table S2). In all locations, collection kits from the company NatureMetrics were used. The sampling method consisted of using a bucket previously sterilized with 90% ethanol and covered with a plastic bag that was changed after each sampling event. First, a 1-L water sample was collected along a linear transect using a 1 L bottle previously cleaned with 90% ethanol, approximately every 50 m. Once 7 L were collected, water filtering was started by attaching a 60 ml sterile syringe to a filter disk of 0.8 µm pore size with a plastic case to prevent contamination of the filter due to manipulation. Once no more water could go through the filter, the syringe was detached, and a smaller syringe with a preserving buffer was used to protect the filter. After each sampling episode, the bucket was cleaned again with ethanol and covered with a new bag in the following sampling episode. All procedures during sampling episodes were done using sterile gloves to prevent contamination with human DNA during sample filtering (Martinelli-Marin et al., 2020; Lozano-Mojica and Caballero, 2021). One location known as “Batallion”, close to Canal del Dique, was used as a positive control for eDNA detections, considering that at least 3 *T. manatus* are kept in semi-captivity in an artificial lake at this site.

Filters were shipped to Nature Metrics (Egham, Surrey, England), where DNA extraction amplification and sequencing took place. DNA from each filter was done using the Qiagen Tissue Mini kit (see manufacturer instructions), modifying some steps to obtain increased DNA yields. Afterward, DNA was purified using the DNeasy PowerClean Pro Cleanup kit to remove PCR inhibitors. DNA obtained from each filter was then amplified using 12 replicated and negative and positive amplification controls, using 12s vertebrate primers (Riaz et al., 2011). Tails were added at the 5′ end of the primers to be complementary with Illumina Nextera index primers. The amplification mixture for each replicate contained 1X DreamTaq PCR Master Mix (Thermo Scientific), 0.4 μM of each of the tailed primers, 1 µL of DNA, and PCR grade water (Thermo Scientific) up to a total reaction volume of 10 µL. All PCRs were run alongside template negatives controls and a mock community of fish species known not to occur in Colombia. PCR conditions consisted of an initial denaturation at 95°C for 2 min, followed by 10 cycles of 20 s at 95°C, a 30 s touchdown annealing step (-0.5°C per cycle) starting at 60°C, and 40 s at 72°C, 35 cycles of 20 s at 95°C, 30 s at 55°C, and 40 s at 72°C, and a final elongation step at 72°C for 5 min.

Success of the amplifications was confirmed via gel electrophoresis. All PCR replicates were pooled and purified using MagBind TotalPure NGS (Omega Biotek) magnetic beads with a ratio 0.8:1 (beads:DNA) to remove primer dimers. Sequencing libraries were prepared from the purified amplicons following a combinational dual index approach (following Illumina’s 16S Metagenomic Sequencing Library Preparation protocol). The libraries were pooled in equimolar concentrations and sequenced on an Illumina MiSeq with a V2 2 × 250 bp kit, the final library was loaded at 12 pM with a 10% PhiX control spike.

Paired-end FASTQ reads for each sample were merged with USEARCH v11 (Edgar, 2010) with a minimum of 80% agreement in the overlap. Forward and reverse primers were trimmed from the merged sequences using cutadapt 2.3 (Martin, 2011) and retained if the trimmed length was between 80 and 120 bp. These sequences were quality filtered with USEARCH to retain only those with an expected error rate per base of 0.01 or below and dereplicated by sample, retaining singletons. Unique reads from all samples were denoised in a single analysis with UNOISE (Edgar, 2016), requiring retained ZOTUs (zero-radius OTUs) to have a minimum abundance of 8. ZOTUs were clustered at 99% similarity with USEARCH. An OTU-by-sample table was generated by mapping all dereplicated reads for each sample to the OTU representative sequences with USEARCH at an identity threshold of 97%.

Consensus taxonomic assignments were made via PROTAX (De Camargo et al., 2017; Axtner et al., 2019) and BLAST (Altschul and Koonin, 1990; Camacho et al., 2009) searches of the representative sequences against the nt database. BLAST hits had a minimum e-score of 1e-20 and a hit length of at least 90% of the query sequence. Identifications from either source were accepted and all cases were consistent at the level at which they were made. Species-level assignments required at least one BLAST hit of ≥99% similarity, and genus-level assignments required a hit ≥95% similarity. Where there were multiple hits meeting these criteria, public GBIF records for Colombia were used to resolve the conflicts where possible. PROTAX assignments with a probability of ≥0.95 were accepted, with species- and genus-level assignments also requiring a supporting hit at ≥99% and ≥95% respectively. OTUs that were ≥99% similar and had similar co-occurrence patterns were combined with LULU (Frøslev et al., 2017) and low abundance detections <0.05% or <10 reads (whichever was the higher) per sample were omitted. OTUs identified above order-level, as well as OTUs identified as originating from human or livestock, were also removed.

## Results

### Mitochondrial DNA Control Region Population Structure and Genetic Diversity

**FIGURE 3:**
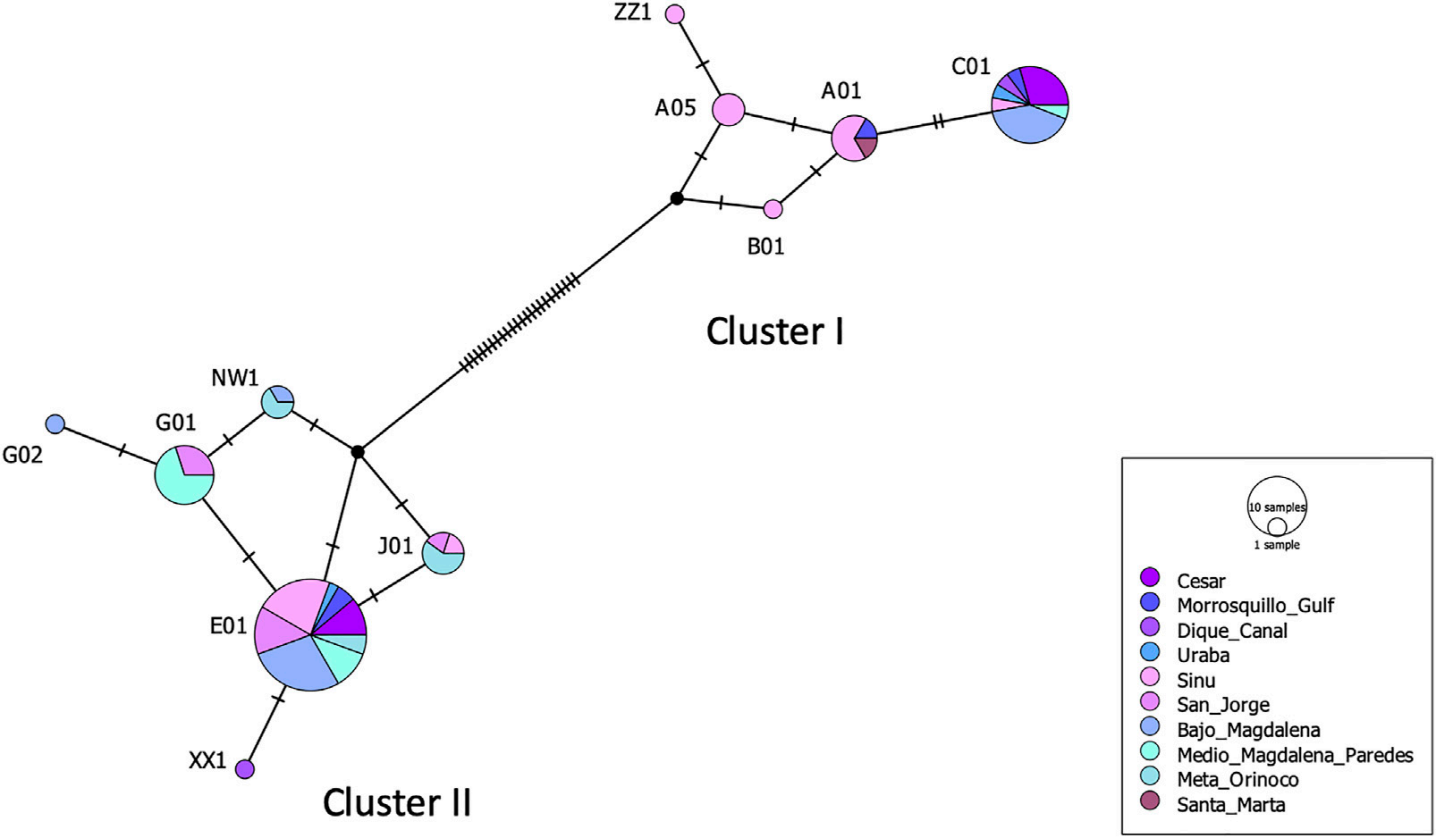
Haplotype network for *Trichechus manatus* from Colombian sampling sites obtained from the TCS analysis. The size of the circle represents the frequency of each haplotype. Cluster I and II refer to haplotype clusters previously defined for *T. manatus* by Vianna et al. (2006) and Satizbal et al. (2012).

**FIGURE 4:**
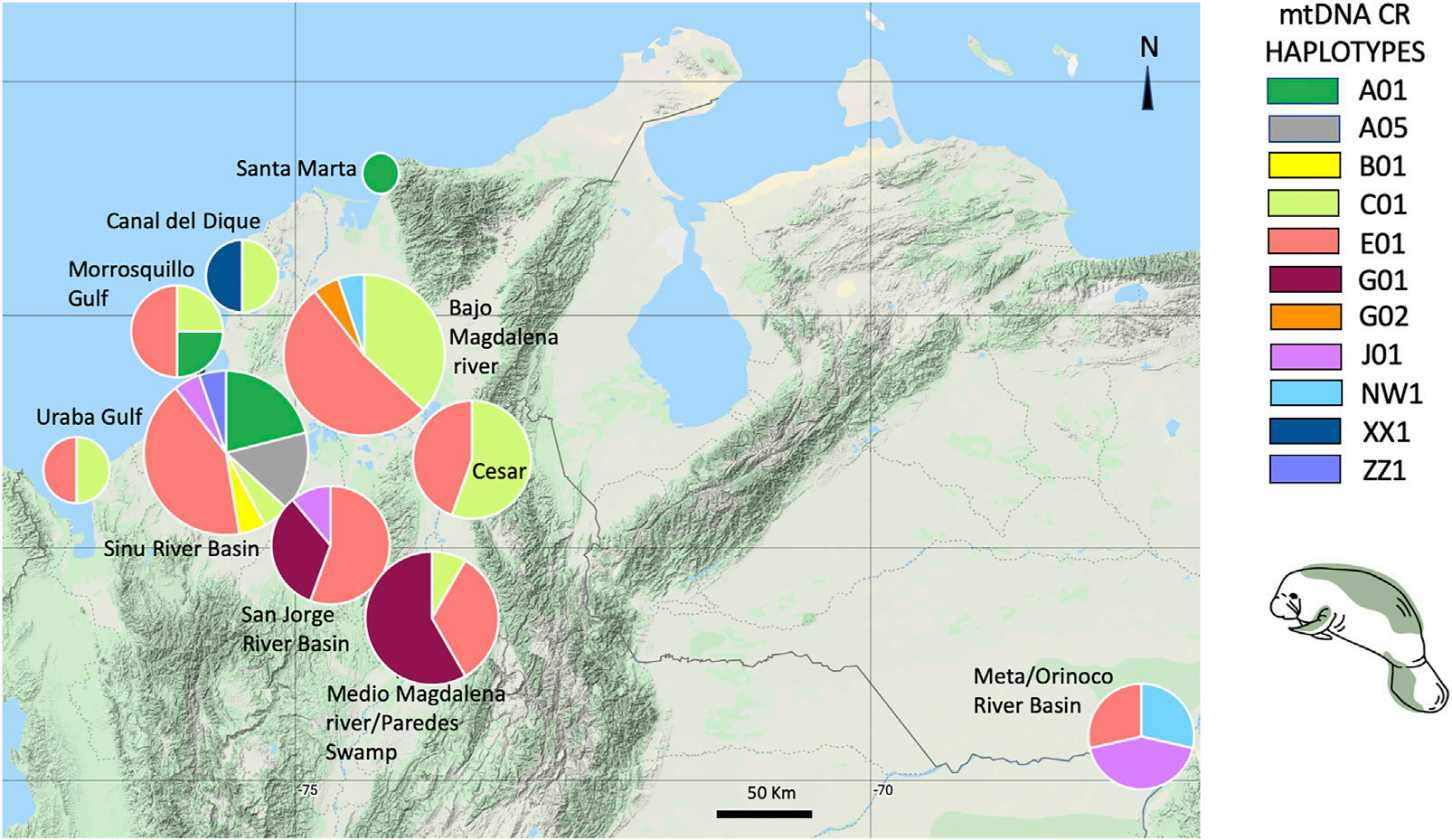
*Trichechus manatus* mtDNA Control region haplotypes and their frequencies found in each Colombian sampling location included in this study.

**FIGURE 5:**
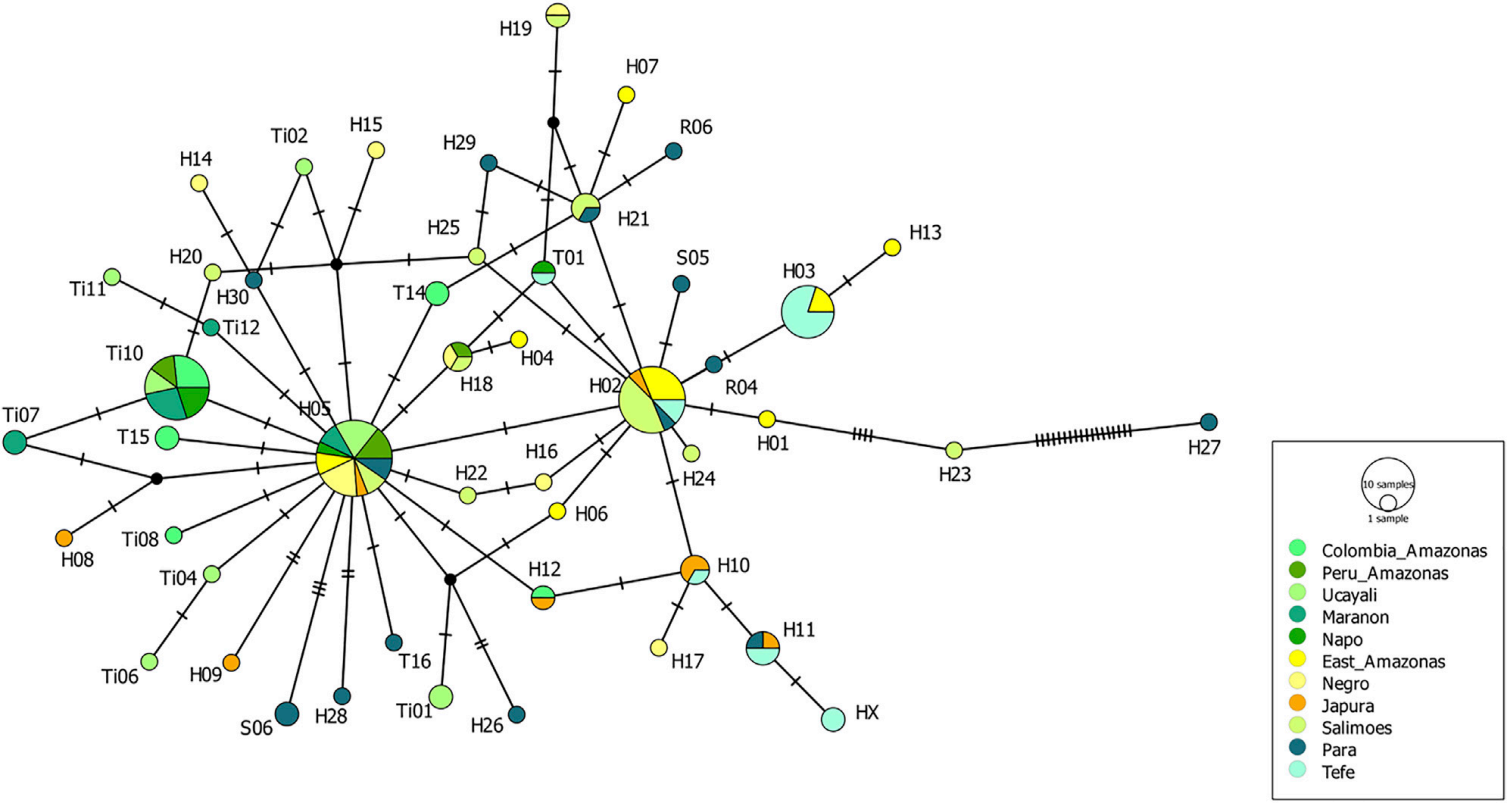
Haplotype network for *Trichechus inunguis* from the Colombian Amazon as well as from the Peruvian and Brazilian Amazon, obtained from the TCS analysis. The size of the circle represents the frequency of each haplotype.

Eleven mtDNA CR haplotypes were identified among a total of 84 *T. manatus* samples collected in Colombian sampling locations (this study and Satizábal et al., 2012). Two new haplotypes were identified in this study (haplotypes XX1 and ZZ1, Genbank accession numbers MZ395961 and MZ395962), identified in Canal del Dique and the Sinú River Basin. In the haplotype network grouping haplotypes found only in Colombian sampling locations (Figure 3), these 11 haplotypes were distributed among two haplotype clusters (I and II), previously identified for *T. manatus* CR (Vianna et al. (2006) and Satizábal et al., 2012). The newly identified haplotypes each belonged in cluster I and cluster II, respectively. Haplotype ZZ1 was found in one new sample from Sinú River Basin, differing by one mutational step from A05, grouped in cluster I, previously identified also in samples from Sinú River Basin (Figure 3). Haplotype XX1, identified in one sample from Canal del Dique, differing by one mutational step from E01, grouped in cluster II. Haplotypes C01 (cluster I) and E01 (cluster II) appear to be two ancestral haplotypes in the Colombian populations. A map of sampled regions with haplotypes for *T. manatus* and their frequency in each of these regions is presented in Figure 4. In the haplotype network built with haplotypes identified from *T. manatus* from Florida, the Caribbean, Central, and South America, haplotypes from Colombia were again distributed among these two clusters, while samples from Brazil and French Guiana grouped in cluster III (Supplementary Figure S1). For the samples from *T. inunguis* included in this study, all four shared the same haplotype Ti10, a common haplotype found from the Colombian Amazon River, the Peruvian Amazon, Ucayali, Marañon, and Napo rivers (Figure 5).

For *T. manatus*, samples from the Santa Marta, Urabá Gulf and Canal del Dique could not be included in the population analysis due to their low sample sizes (n ≤ 2). Significant population differentiation at the *F*_*st*_ level was found among Sinú River Basin and Cesar Province, Medio Magdalena River and Bajo Magdalena River; among Cesar Province and Magdalena Medio River and Meta/Orinoco rivers basins; among Bajo Magdalena River and Medio Magdalena River and Meta/Orinoco river basins and between Magdalena Medio River and Meta/Orinoco river basins. At the *Φ*_*st*_ level, differentiation was found among Cesar Province and Magdalena Medio River, San Jorge River Basin, and Meta/Orinoco river basins; between Gulf of Morrosquillo and Magdalena Medio River and between San Jorge River Basin and Bajo Magdalena River (Table 1). When the AMOVA analysis included populations from Florida, the Caribbean, Central, and South America, significant differentiation was found among almost all Colombian locations and these populations, both at the *F*_*st*_ and *Φ*_*st*_ levels (Supplementary Table S3). Lack of differentiation at the *F*_*st*_ level was found among Morrosquillo Gulf and the Dominican Republic, Chetumal Mexico, and Guyana and among Meta/Orinoco river basins and Chiapas Mexico and Chetumal Mexico. Lack of differentiation at the *Φ*_*st*_ level was found between Cesar Province and Chetumal Mexico, among Morrosquillo Gulf and Chetumal Mexico and Guyana, between Sinú River Basin and Chetumal Mexico, and between Meta/Orinoco river basins and Chiapas Mexico. For *T. inunguis*, significant population differentiation was found at both the *F*_*st*_ and *Φ*_*st*_ levels between the Colombian Amazon River and all locations from the Brazilian Amazon. However, lack of differentiation was found between the Colombian Amazon River and most locations in the Peruvian Amazon, including the Peruvian Amazon River, Napo and Marañon rivers (Table 2).

**TABLE 1:** Pairwise *Fst* (below diagonal) and *Φ*_*st*_ (above diagonal) values for the mitochondrial control region (410 bp) among 7*T. manatus* Colombian geographic locations. Three locations (Santa Marta, Canal del Dique and Urabá Gulf) were excluded from the analysis because of their small sample size.

*Φ*_*st*_ *F*_*s t*_	Cesar province (n = 9)	Morrosquillo Gulf (*n* = 4)	Sinú River basin (*n* =19)	San Jorge River basin (*n* = 9)	Bajo Magdalena River (*n* = 19)	Medio Magdalena/Paredes Marsh (*n* = 12)	Meta/Orinoco River basins (*n* = 7)
Cesar Province	−	0.199	0.047	**0.490**	0.015	0.372	0.432
Morrosquillo Gulf	0.058	—	0.166	**0.510**	0.129	0.316	0.421
Sinú River Basin	0.160	0.092	—	**0.389**	0.013	0.306	0.348
San Jorge River Basin	0.207	0.001	0.057	—	**0.235**	0.019	0.043
Bajo Magdalena River	0.231	0.053	**0.079**	0.116	—	0.145	0.197
Medio Magdalena/Paredes Marsh	0.326	0.181	**0.188**	0.010	**0.240**	—	0.004
Meta/Orinoco river basins	0.253	0.075	0.097	0.121	**0.191**	0.264	—

Probability values are based on 10,000 permutations. Significantly different values (*p* < 0.05) in bold.

**TABLE 2:** Pairwise *Fst* (below diagonal) and *Φ*_*st*_ (above diagonal) values for the mitochondrial control region (361 bp) among 11 *T.inunguis* geographic locations.

*Φ*_*st*_ *F*_*s t*_	Colombian Amazon River (*n* = 10)	Peru Amazon River (*n* = 6)	Ucayali River (Peru) (*n* = 12)	Marañon River (Peru) (*n* = 9)	Napo River (Peru) (*n* = 5)	Eastern Amazon River (Brazil) (*n* = 14)	Negro River (Brazil) (*n* = 10)	Japura River (Brazil) (*n* = 8)	Salimoes River (Brazil) (*n* = 18)	Para (Brazil) (*n* = 16)	Tefe (*n* = 16) (Brazil)
Colombian Amazon River	—	0.038	0.052	0.058	0.009	**0.319**	**0.089**	**0.209**	**0.234**	**0.069**	**0.224**
Peru Amazon River	0.098	—	0.022	0.055	0.089	**0.300**	0.013	**0.208**	**0.199**	0.029	**0.198**
Ucayali River	**0.088**	0.043	—	**0.132**	0.061	**0.267**	**0.051**	**0.187**	**0.219**	**0.070**	**0.192**
Marañon River	0.027	0.022	0.026	—	0.035	**0.419**	**0.216**	**0.327**	**0.329**	**0.136**	**0.346**
Napo River	0.009	0.025	0.038	0.077	—	**0.258**	0.052	**0.194**	**0.162**	0.022	**0.202**
East Amazon River	**0.154**	**0.127**	**0.083**	**0.147**	**0.175**	—	**0.097**	**0.133**	0.003	0.022	**0.111**
Negro River	**0.156**	0.029	0.007	**0.097**	0.135	**0.080**	—	0.043	0.036	0.010	0.043
Japura River	**0.098**	0.088	0.041	**0.106**	**0.132**	0.026	0.038	—	**0.099**	0.002	0.026
Salimoes River	**0.163**	**0.143**	**0.103**	**0.162**	**0.189**	0.016	**0.092**	0.038	—	0.010	**0.072**
Para	**0.092**	**0.066**	**0.027**	**0.087**	**0.109**	0.035	0.023	0.006	**0.041**	—	0.017
Tefe	**0.222**	0.016	0.030	**0.147**	**0.182**	**0.090**	0.002	0.052	**0.111**	**0.064**	—

Probability values are based on 10,000 permutations. Significantly different values (*p* < 0.05) in bold.

For *T. manatus*, haplotype diversity (*h*) was highest for Canal del Dique and Urabá Gulf, with one haplotype defined for each of the two samples in each location. Morrosquillo Gulf also showed high haplotype diversity, followed by Sinú River Basin and Meta/Orinoco river basins. San Jorge River Basin, Medio Magdalena, and Bajo Magdalena River had similar but slightly lower levels of haplotype diversity, and Cesar Province showed the lowest haplotype diversity (Table 3). Nucleotide diversities (*π*) were highest for Canal del Dique and Urabá Gulf, followed by Morrosquillo Gulf, Cesar Province, Sinú River Basin, and Bajo Magdalena River, with the lowest values found in Medio Magdalena River and Meta/Orinoco river basins. When haplotype and nucleotide diversities were compared with those of other locations from Florida, the Caribbean, Central, and South America, the higher values found in Colombian locations were similar to those found in Placencia Belize, Chetumal Mexico, and French Guiana (Supplementary Table S4). For *T. inunguis*, haplotype (*h*) diversity was similar among the Colombian Amazon River and the Eastern Amazon River, Negro River, Salimoes River, and Tefé and slightly lower than the value for Japura River (Table 4). Nucleotide diversities (*π*) were similar among the Colombian Amazon River and the Eastern Amazon River, the Napo River and Tefé and slightly lower than Ucayali River, Negro River, and Japura River (Table 4).

**TABLE 3:** Genetic diversity statistics for *T. manatus* from Colombian sampling locations. Haplotype diversity (*h*) and nucleotide (*π*) diversity (in %) are shown for each geographic location (±SD). The Santa Marta location was excluded due to small sample size (*n* = 1).

	Cesar province	Morrosquillo Gulf	Canal del Dique	Urabá Gulf	Sinú River basin	San Jorge River basin	Bajo Magdalena River	Medio Magdalena/Paredes Marsh	Meta/Orinoco River basins
No. individuals	9	4	2	2	19	9	19	12	7
No. Haplotypes	2	3	2	2	7	3	4	3	3
*h*	0.556 ± 0.090	0.833 ± 0.222	1.000 ± 0.500	1.000 ± 0.500	0.784 ± 0.074	0.639 ± 0.126	0.614 ± 0.075	0.591 ± 0.108	0.762 ± 0.115
π	3.6 ± 0.020	4.5 ± 0.030	6.8 ± 0.069	6.6 ± 0.067	3.5 ± 0.018	0.2 ± 0.002	3.3 ± 0.017	1.2 ± 0.007	0.4 ± 0.003

**TABLE 4:** Genetic diversity statistics for *T. inunguis*. Haplotype diversity (*h*) and nucleotide (*π*) diversity (in %) are shown for each geographic location (±SD).

	Colombian Amazon River	Peru Amazon River	Ucayali River (Peru)	Maranon River (Peru)	Napo River (Peru)	Eastern Amazon River (Brazil)	Negro River (Brazil)	Japura River (Brazil)	Salimoes River (Brazil)	Para (Brazil)	Tefe (Brazil)
No. Individuals	10	6	12	9	5	14	10	8	18	16	16
No. Haplotypes	5	3	7	4	3	8	7	7	10	14	6
*H*	0.822 ± 0.120	0.733 ± 0.155	0.002 ± 0.002	0.778 ± 0.110	0.700 ± 0.218	0.868 ± 0.076	0.867 ± 0.107	0.964 ± 0.077	0.850 ± 0.077	0.983 ± 0.028	0.742 ± 0.104
*Π*	0.5 ± 0.003	0.2 ± 0.002	0.6 ± 0.003	0.3 ± 0.002	0.4 ± 0.003	0.5 ± 0.003	0.7 ± 0.005	0.7 ± 0.004	0.6 ± 0.004	1.6 ± 0.009	0.5 ± 0.003

### *T. manatus* and *T. inunguis* eDNA Detection

Water samples were successfully collected from 21 locations in Colombia to try detection for both species. *T. manatus* eDNA was successfully detected in seven filters from four locations. The majority of detections occurred in the Canal del Dique, with three detections, followed by two detections in the Batallion (positive control), one in the Lorica Marsh (Sinú River Basin), and one in the mouth of the Atrato River. The number of *T. manatus* eDNA reads ranged between 57 and 737 reads per filter, with the highest number of reads obtained from the Batallion location (Table 5). No *T. manatus* detections were obtained for Ayapel Marsh (San Jorge River Basin) or the Orinoco River Basin. The reads identified as *T. manatus* in the dataset were identical (100% similarity, 100% coverage) to those 12s RNA *T. manatus* sequences previously deposited on Genbank with accession numbers KX381539.1, AM904728.1, AY012104.1, and U60183.1. The percentage of reads for the target species compared to the total number of reads in each filter, was included in Table 5.

**TABLE 5:** Positive *T. manatus* eDNA detections in filters collected in four out of 18 possible Colombian locations. The target sequence was the hyper variable 12sRNA gene in the mtDNA. The total of reads detected in each filter is also showed as well as the percentage corresponding to the *T. manautus manatus* reads regarding the total for the filter. Filter numbers corresponds here to the *n*th positive filter for the location rather than to the correct number of said filter.

Sampling location	Total number of *T. manatus* reads	Target reads/Total reads filter 1 (%)	Target reads/Total reads filter 2 (%)	Target reads/Total reads filter 3 (%)
Canal del Dique	307	82/49,958 (0.16%)	168/33,834 (0.49%)	57/78,455 (0.07%)
Batallion (positive control)	802	802/28,812 (2.56%)	—	—
Lorica Marsh (Sinu River basin)	161	161/83,032 (0.19%)	0	0
Atrato River mouth (Atrato river basin)	159	159/5,750 (2.76%)	0	0

The negative control had a very low total number of reads 2273) of which 2081 corresponded to human DNA, and the remaining 192 reads were split across 51 OTUs, with seven OTUs having counts between 10 and 18 reads and the rest had fewer than 10 reads. In all cases the number of reads found in the samples was higher than in the negative control after the per-sample filter of 0.05% and a minimum of 10 reads applied in the filtering process.

Unfortunately, no *T. inunguis* detections were obtained in the three sampling locations in the Colombian Amazon River.

## Discussion

### Confirmation of Previous Results for *T. manatus* and Initial Results from New Sampling Areas

This study presents new information about the mitochondrial population structure and the genetic diversity of Antillean and Amazonian manatees in Colombia. As more regions are accessible after the peace process signed between the Colombian government and The Revolutionary Armed Forces of Colombia—People’s Army (in Spanish: Fuerzas Armadas Revolucionarias de Colombia—Ejército del Pueblo, FARC) in 2016, new and additional information is becoming available about wildlife populations in this region. This is the case for populations of Antillean manatees in the Urabá Gulf and the Atrato River Basin, from which little or no information was known. Also, new opportunities for starting monitoring and conservation programs in these new areas becomes possible, and this is the reason why detection of manatees via eDNA analyses is a fast a low cost alternative to define them.

These new analyses confirm population structure patterns previously described, including significant differentiation between Antillean manatee populations from the Bajo Magdalena and Magdalena Medio river basins, between Magdalena River and the Meta/Orinoco river basins, and between Sinú and the Magdalena rivers (Satizábal et al., 2012). The small sample size from the Morrosquillo Gulf possibly prevented significant differentiation between this sampling location and the other sampling locations at the *F*_*st*_ level. However, at the *Φ*_*st*_ level, significant differentiation between Morrosquillo Gulf and Magdalena Medio River was evidenced, possibly due to the presence of one haplotype (A1) that has not been found in the Magdalena River. The lack of significant differentiation at the *F*_*st*_ level between San Jorge River Basin and both Sinú and Magdalena rivers (including the sampling location in Cesar province) could further confirm the potential for connectivity between these two basins via flooding plains (i.e., marshes in the San Jorge River Basin and in the Magdalena River Basin) or by connectivity maintained by animals moving from the Sinu River to the coast via the Canal del Dique to the Bajo Magdalena and San Jorge River, as suggested in Satizábal et al. (2012). Global genetic differentiation analyses confirmed and supported differentiation of the Colombian populations with population from most of the other geographic locations in Florida, the Caribbean, Central, and South America. Lack of differentiation between some sampling locations may be a likely result of sharing some haplotypes, such as haplotypes A01, C01, and E01, haplotypes that appear to be ancestral and widespread in populations from Central America, Colombia, and the Caribbean. Mitochondrial genetic diversity appears to be high for most Colombian sampling locations and similar to the levels found in French Guiana and Mexico, confirming results from Vianna et al. (2006) and Satizábal et al. (2012).

Interestingly, one new haplotype (XX1) was found in one of the samples collected in Canal del Dique, one of the new locations sampled in this study. The Canal del Dique was initially constructed in the XVI century to connect the Magdalena River and its marsh with the Bay of Cartagena (Benitez Hurtado, 2017). Over the years, it has become one of the main freshwater inputs into the Bay of Cartagena, and due to the high load of sediments carried by the Magdalena River (Restrepo et al., 2014), the Canal has almost lost its navigability and is one of the main sources of pollutants affecting the Bay and the marshes in this area (Tosic et al., 2019). It is then surprising to still find manatees in the Canal, detected both by direct observation and with successful eDNA detections, and currently, conservation efforts are undergoing in this area with the help of the local communities (Fundación Omacha, pers. comm). Also, access to new sampling regions such as the Urabá Gulf allows us to have a clearer picture of the levels of genetic diversity found in the Antillean Colombian manatee populations.

### New population structure patterns for *T. inunguis* and the importance of female mediated gene flow

For the Amazonian manatees, even by increasing the sample sizes for the Colombian Amazon River by three samples, patterns regarding populations structure appear to change, suggesting more genetic similarity between the Colombian Amazon River groups and those from the Peruvian Amazon River and its tributaries, including the Marañon, Ucayali and Napo rivers and suggesting significant mitochondrial genetic differentiation between the Colombian Amazon River and the Eastern Amazon River and its tributaries in the Brazilian Amazon, contradicting some of the findings by Satizábal et al. (2012). A similar pattern of populations differentiation between the western and eastern Amazon River Basin was found for the riverine dolphin *Sotalia fluviatilis* (Caballero et al., 2018). These results also support female mediated gene flow in the rivers in the Western Amazon Basin, which is a relevant result for conservation management programs, in which maintaining this connectivity must be one of the goals. Obstacles to connectivity due to dam and hydroelectric construction may have a devastating effect on Amazonian manatee populations (Arraut et al., 2017).

### Use of eDNA for Antillean and Amazonian Manatee Detection in Colombia

This study is, to our knowledge, the first time eDNA analyses are used to detect both manatee species in Colombian rivers, marshes, and bays. Positive detection results were found for four out of 18 sampling locations for *T. manatus*, including one location used as a positive control since manatees were known to inhabit a small lake in the location named Batallion, and no positive detections were obtained for *T. inunguis* in any of the three sampling locations tried. Thus, although detection rates were low, this is still a promising technique to continue using to detect these species in the country. Interestingly, some of the areas where eDNA results were positive, for example, the mouth of the Atrato River and the Canal del Dique, are areas in which little to no manatee studies have been developed. These positive results could then indicate areas in which additional research should be undertaken and areas where conservation initiatives with the local communities could be started.

The lack of positive detections in other sampled areas could be related to many factors, including environmental factors, such as the water pH. It is known that DNA tends to degrade at a faster rate in an acidic environment (Seymour et al., 2018). Some of the lakes sampled in this study in the Amazon are known as “blackwater” lakes (i.e., Tarapoto and Caballo Cocha). Blackwaters are characterized by low pH and high tannin content, which may affect the persistence of eDNA in these lakes. The season in which samples were collected, the low water season, may also have influenced our detection success since manatees could have moved away from marshes and lakes into deeper waters in the main river channels. Samples were collected superficially, at a maximum of 1-m depth. If the animals stay in areas with higher depths, this may also be a factor affecting the detection rate. A low abundance of Amazonian manatees may also reduce the likelihood of finding their DNA in the water (Goldberg et al., 2016). In this case, maybe a different methodology, using species-specific primers as well as a methodology aimed at detecting minimum amounts of eDNA in the sample, for example, qPCR and digital-droplet PCR (ddPCR), could help improve our detection abilities for this particular species (Baker et al., 2018; Hunter et al., 2018). Interestingly, in the Amazon and Orinoco samples we detected eDNA from other aquatic mammals, including river dolphins *Inia geoffrensis* (Martinelli-Marin et al., 2020), tucuxi dolphins *Sotalia fluviatilis* (Martinelli et al., in review) and giant river otters *Pteronura brasiliensis* (Martinelli-Marin et al., 2020).

Nevertheless, eDNA detection appears to be a promising technique that could still be used for manatee detection in Colombia, and continued improvements to the technique can be made to improve detection rates. Also, it would be possible to develop a comparison data set of 12 s sequences from the manatee samples currently available, in order to be able to define presence of particular 12 s haplotypes in areas sampled via eDNA, as has been done for other aquatic organisms such as marine invertebrates (Shum and Palumbi, 2021). Currently, we cannot achieve this. Since we don´t currently have 12 s manatee sequences from Colombia, we cannot conduct direct comparisons with our eDNA data, since PCR artifacts and sequencing errors could overestimate the number of 12 s haplotypes found via metabarcoding, as has been show in other studies (Elbrecht et al., 2018).

Another positive promise of eDNA analyses is its potential to be used in citizen science projects with communities living close to manatee populations. Community involvement would allow sampling in areas that are difficult to access and/or continued sampling for monitoring purposes. Also, their involvement in water sample collecting and filtering could improve community appreciation for manatees and promote their protection.

### Conservation Implication of This Study

Results from this study confirm the need to continue the genetic monitoring of manatee populations in Colombia, particularly when animals are to be released back to their natural habitat since significant mitochondrial genetic differentiation exists among *T. manatus* populations in the main river basins in the country and new mitochondrial haplotypes are still being found. Although ideally, information from nuclear markers should also be considered for these animals, amplification, and sequencing of the mitochondrial Control Region is a cheap and fast alternative for environmental authorities and NGOs to undertake to obtain initial genetic information useful for the conservation programs. For *T. inunguis*, it is key to maintain connectivity among the Amazon River channels and tributaries in the Western Amazon region, and planned dam and hydroelectric construction projects should be carefully evaluated to prevent loss of manatee population connectivity. Environmental DNA detection is emerging as a promising technique to define areas in the country where new conservation initiatives could be undertaken, and an interesting technique to be included in citizen science projects to be developed with local communities in areas of manatee distribution.

## Data Availability

The datasets presented in this study can be found in online repositories. The names of the repository/repositories and accession number(s) can be found below: https://www.ncbi.nlm.nih.gov/genbank/, MZ395961: https://www.ncbi.nlm.nih.gov/genbank/, MZ395962. Raw data (reads) were made available in DRYAD Repository (https://datadryad.org/stash/share/Qtb2EFuCHoZdUSrMnKu6m2FeWJ01x1_mL0IO96mkauA).
