# Supplementary figures and images for: Mitochondrial Genetic Diversity, Population Structure and Detection of Antillean and Amazonian Manatees in Colombia: New Areas and New Techniques
Source: Front Genet. 2021 Nov 26;12:726916. doi: 10.3389/fgene.2021.726916 (PMC8662808; doi:10.3389/fgene.2021.726916)

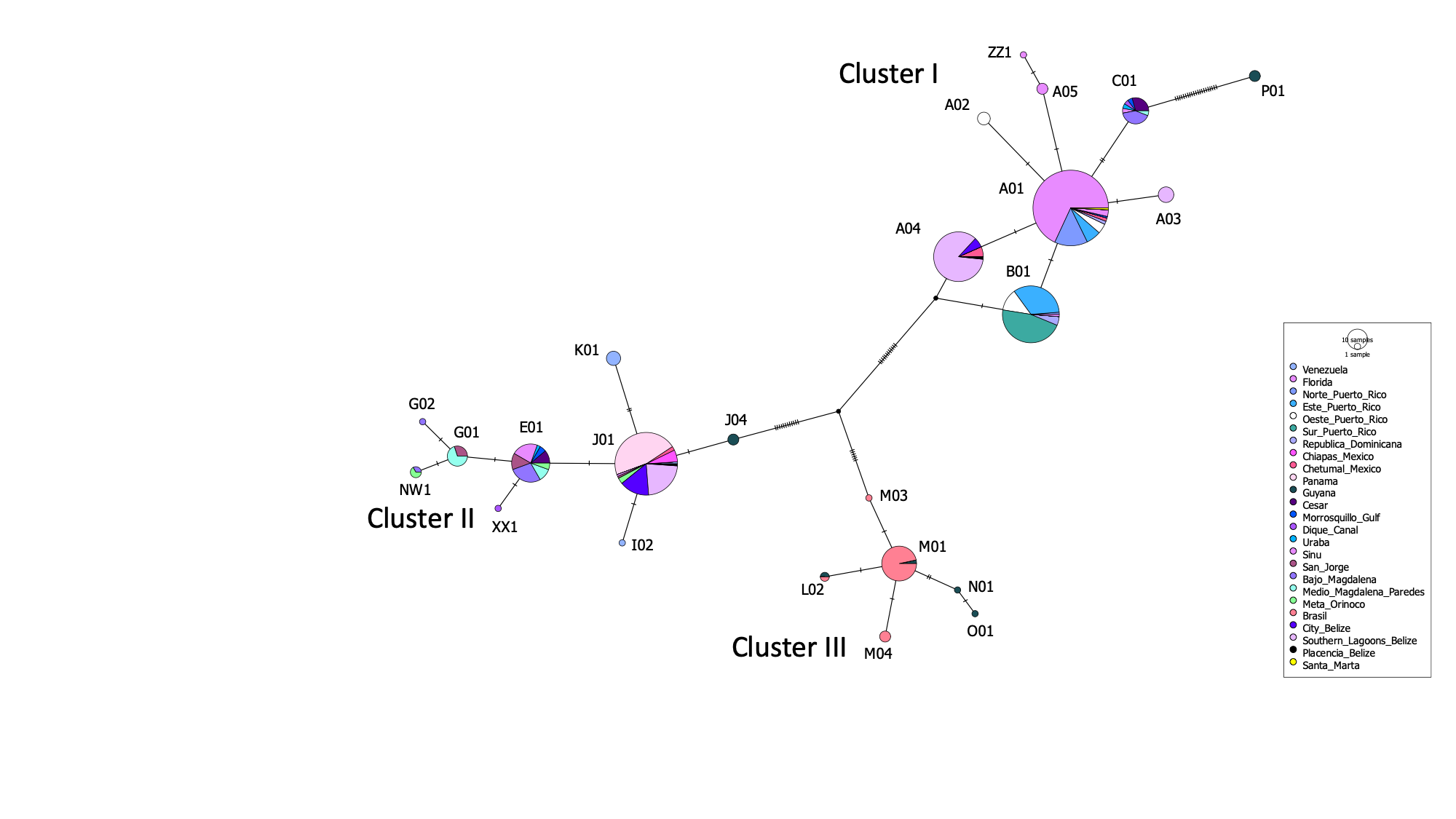

Supplement: Supplementary file 1 [file Image1.TIFF]
