# Supplementary material for: Mitochondrial Genetic Diversity, Population Structure and Detection of Antillean and Amazonian Manatees in Colombia: New Areas and New Techniques
Source: Front Genet. 2021 Nov 26;12:726916. doi: 10.3389/fgene.2021.726916 (PMC8662808; doi:10.3389/fgene.2021.726916)
Supplement: Supplementary file 3 [file Table1.DOCX]

**Supplementary Table 1.** Manatee tissue sampling sites (new to this study). Refer to Figure 1 for locations in the map.

| **Sampling site** | **Geographic region or river basin** | **Number of samples (This study)** | **Samples from Satizábal et al. (2012)** | **Total number of samples** |
| --- | --- | --- | --- | --- |
| 1. Totumo Marsh, Yondo, Antioquia Province | Medio Magdalena/Paredes Marsh | 1 | - | 1 |
| 1. San Silvestre Marsh, Barranca, Santander Province | Medio Magdalena/Paredes Marsh | 2 | - | 2 |
| 1. Paredes Marsh, Santande Province | Medio Magdalena/Paredes Marsh | 5 | 3 | 8 |
| 1. Aguachica, Cesar Province | Cesar Province | 9 | - | 9 |
| 1. Magangué, Bolivar Province | Bajo Magdalena | 1 | 18 | 19 |
| 1. Canal del Dique, Bolivar Province | Canal del Dique | 2 | - | 2 |
| 1. El Corchal, Bolivar | Morrosquillo Gulf | 1 | - | 1 |
| 1. Cispata Bay, Córdoba Province | Morrosquillo Gulf | 3 | - | 3 |
| 1. Lorica Marsh, Córdoba Province | Sinu basin | 6 | 13 | 19 |
| 1. Ayapel Marsh, Córdoba Province | San Jorge basin | 1 | 8 | 9 |
| 1. Necocli, Antioquia Province | Uraba Gulf | 2 | - | 2 |
| 1. Santa Marta, Magdalena Province | Caribbean Coast | 1 | - | 1 |
| 1. Meta River, Vichada Province | Meta/Orinoco basin | 2 | 5 | 7 |
| 1. Puerto Nariño, Amazonas Province | Colombian Amazon | 3 | 6 | 9 |
