# Supplementary material for: Mitochondrial Genetic Diversity, Population Structure and Detection of Antillean and Amazonian Manatees in Colombia: New Areas and New Techniques
Source: Front Genet. 2021 Nov 26;12:726916. doi: 10.3389/fgene.2021.726916 (PMC8662808; doi:10.3389/fgene.2021.726916)
Supplement: Supplementary file 4 [file Table2.DOCX]

**Supplementary Table 2.** Environmental DNA sampling sites. Refer to Figure 2 for locations in the map.

| **Sampling location** | **Geographic region of river basin** | **Number of filters** |
| --- | --- | --- |
| 1. Chucuri Marsh | Medio Magdalena/Paredes Marsh | 3 |
| 1. San Juan river | Medio Magdalena/Paredes Marsh | 3 |
| 1. Paredes Marsh | Medio Magdalena/Paredes Marsh | 4 |
| 1. De las Flores Marsh | Canal del Dique | 2 |
| 1. Canal del Dique | Canal del Dique | 1 |
| 1. El Floral Marsh | Canal del Dique | 2 |
| 1. Batallion lake (positive control) | Canal del Dique | 1 |
| 1. Lorica Marsh | Sinu river basin | 3 |
| 1. Cispata Bay | Morrosquillo Gulf | 3 |
| 1. Ayapel Marsh | San Jorge river basin | 3 |
| 1. Atrato river mouth | Atrato river basin | 3 |
| 1. Suriqui river mouth | Uraba Gulf | 4 |
| 1. Marriaga Marsh | Atrato river basin | 4 |
| 1. Rio Negro cove | Uraba Gulf | 1 |
| 1. Meta river | Meta/Orinoco basin | 3 |
| 1. Vita river | Meta/Orinoco basin | 3 |
| 1. Orinoco river | Meta/Orinoco basin | 3 |
| 1. Bojonawi reserve | Meta/Orinoco basin | 3 |
| 1. Puerto Nariño | Amazon river basin | 3 |
| 1. Tarapoto Lake | Amazon river basin | 3 |
| 1. Caballo Cocha Lake | Amazon river basin | 3 |
