# Supplementary material for: Mitochondrial Genetic Diversity, Population Structure and Detection of Antillean and Amazonian Manatees in Colombia: New Areas and New Techniques
Source: Front Genet. 2021 Nov 26;12:726916. doi: 10.3389/fgene.2021.726916 (PMC8662808; doi:10.3389/fgene.2021.726916)
Supplement: Supplementary file 5 [file Table4.DOCX]

**Supplementary Table 4**. Genetic diversity statistics for *T.manatus*. Haplotype diversity (h) and nucleotide (π) diversity (in %) are shown for each geographic location (±SD).

|  | Venezuela | Florida | Puerto Rico  North | Puerto Rico  South | Puerto Rico  East | Puerto Rico  West | Dominican Republic | Chiapas Mexico | Chetumal Mexico | Panama | French Guiana | Brazil | City Belize | Southern Lagoons Belize | Placencia Belize |
| --- | --- | --- | --- | --- | --- | --- | --- | --- | --- | --- | --- | --- | --- | --- | --- |
| No. individuals | 7 | 96 | 21 | 37 | 36 | 20 | 6 | 6 | 8 | 45 | 10 | 34 | 19 | 80 | 2 |
| No. Haplotypes | 3 | 1 | 2 | 1 | 2 | 3 | 2 | 1 | 3 | 1 | 6 | 4 | 2 | 3 | 2 |
| h | 0.524  ±  0.209 | 0 | 0.095  ± 0.084 | 0 | 0.386 ± 0.074 | 0.6526 ± 0.0648 | 0.533  ±  0.172 | 0 | 0.714  ±  0.123 | 0 | 0.867  ±  0.137 | 0.271  ± 0.096 | 0.351 ± 0.111 | 0.502  ±  0.046 | 1 |
| π | 0.3  ±  0.002 | 0 | .02  ±  0.0005 | 0 | 0.09 ± 0.001 | 0.2 ± 0.002 | 0.1  ±  0.001 | 0 | 2.8  ± 0.016 | 0 | 4.7  ± 0.026 | 0.06  ± 0.0008 | 2.2  ± 0.012 | 2.6  ± 0.013 | 6.3  ±  0.064 |
